# Corticosteroid Therapy in Dengue Infection- Opinions of Junior Doctors

**DOI:** 10.4103/0974-777X.62861

**Published:** 2010

**Authors:** Senaka Rajapakse, Champa Ranasinghe, Chathuraka Rodrigo

**Affiliations:** *Department of Clinical Medicine, Faculty of Medicine, University of Colombo, Colombo, Sri Lanka.*; 1 *University Medical Unit, National Hospital, Colombo, Sri Lanka.*

Sir,

Infection with dengue virus imperils approximately 20 million people every year in tropical and sub tropical countries.[1] Improved management protocols have resulted in a large decrease in mortality, which is approximately one to two per cent.[1] The incidence of dengue is high in Sri Lanka; during the 2004 epidemic, 15457 cases with 88 deaths were reported;[2] in 2008, 6529 patients were reported. The World Health Organization (WHO) has published guidelines for the management of dengue infection,[1] available to hospitals in Sri Lanka. These guidelines were based largely on pediatric practice. In addition, the Ministry of Health, Sri Lanka, has published guidelines on the management of dengue.[3] Corticosteroids are used in practice by clinicians for various manifestations of dengue such as shock syndrome, thrombocytopenia and special situations, despite the lack of evidence of benefit. The WHO guidelines do not mention the use of corticosteroids, and the Sri Lankan guidelines state that corticosteroids are ineffective in preventing shock in dengue hemorrhagic fever, and may even be harmful by causing gastro-intestinal bleeding, although this recommendation was not based on trial evidence. Trial evidence on the use of steroids in dengue is very limited[4] and the need for further studies has been recommended. A survey of physicians and pediatricians in Sri Lanka showed that the majority (71%) did not consider steroid therapy beneficial.[5] We conducted this survey to determine the knowledge and practices of non-specialist grade doctors, in general medical wards of a tertiary care hospital, regarding the use of corticosteroids in dengue infection.

A self administered questionnaire was given to all non-specialist grade doctors working in general medical wards of the National Hospital, Colombo, Sri Lanka, in December, 2008. The National Hospital is the largest tertiary care hospital in Sri Lanka, located in the capital city of Colombo. Medical officers who had completed internship and served in the capacity of senior house officers (SHOs), registrars and senior registrars were included in the sample. Ethical clearance for the study was granted by the Ethics Review Committee of the National Hospital. Questions covered opinions of doctors on corticosteroid use in different complications of dengue; whether corticosteroids are likely to be harmful, and whether there was a need for a randomized controlled trial on corticosteroids in dengue.

The sample included 43 doctors. There were eight (18.6%) SHOs, six (14%) senior registrars and 29 (67.4%) registrars in the sample. Thirty three (76.7%) doctors stated that they manage patients on clinical judgment, while seven (16.3%) said they adhere to WHO guidelines when managing dengue fever. Two (4.7%) respondents claimed dependence on clinical judgment as well as guidelines while one preferred local guidelines over WHO guidelines. The majority of the doctors (37, 86%) were unsure about benefits of corticosteroids in treating thrombocytopaenia. One (2.3%) thought corticosteroids would reduce the rate of drop in the platelet count, and five (11.6%) thought that there would be definitely no benefit. On steroids reducing the risk of shock, five (11.6%) said yes, four (9.3%) said no and 34 (79.1%) were not sure. Ten (23.3%) respondents thought corticosteroids were of no use but were harmless in the treatment of dengue while two (4.7%) said corticosteroids were harmful. The rest (31, 72.1%) were unsure as to whether treatment with corticosteroids was harmful. Forty (93%) doctors agreed on the need for a randomized controlled clinical trial on use of steroids in dengue fever. Three (seven per cent) said it was not necessary. Only four (9.3%) doctors in the sample had used steroids in dengue fever previously.

Many non-specialist grade doctors appear to use clinical judgment rather than published guidelines in the management of dengue. Most of them were unsure of corticosteroid use in treatment of dengue. It is likely that practices with regard to the use of corticosteroids in dengue vary widely. One clear deficit is that the current guidelines do not give a clear recommendations regarding the place of corticosteroids in dengue, and this is largely due to the lack of convincing evidence for or against its benefits. The majority of non-specialist grade doctors felt the need for further studies on benefits of corticosteroids in dengue, and we support this view. A randomized controlled trial on the efficacy of corticosteroids in treating various manifestations of dengue is planned.

## References

[1] WHO (1997). Dengue haemorrhagic fever: Diagnosis, treatment, prevention and control.

[2] Epidemiology Unit Disease situation report.

[3] Guidelines on Clinical Management of Dengue Fever / Dengue HaemorrhagicFever. Epidemiology Unit.

[4] Rajapakse S (2009). Corticosteroids in the treatment of dengue illness. Trans R Soc Trop Med Hyg.

[5] Kularatne SA (2005). Survey on the management of dengue infection in Sri Lanka: Opinions of physicians and pediatricians. Southeast Asian J Trop Med Public Health.

